# Cross-walk of the Chronic Liver Disease Questionnaire for Nonalcoholic Steatohepatitis (CLDQ-NASH) and the EuroQol EQ-5D-5L in patients with NASH

**DOI:** 10.1186/s12955-023-02195-x

**Published:** 2023-10-14

**Authors:** Jesse Fishman, Victoria Higgins, James Piercy, James Pike

**Affiliations:** 1 Madrigal Pharmaceuticals, Conshohocken, PA USA; 2 Adelphi Real World, Bollington, UK

**Keywords:** CLDQ-NASH, EQ-5D-5L, Cross-walk analysis, Nonalcoholic steatohepatitis, Patient-reported outcome measures

## Abstract

**Background:**

Nonalcoholic steatohepatitis (NASH) is a chronic progression of nonalcoholic fatty liver disease, which can negatively impact the health-related quality of life (HRQoL) of affected individuals. HRQoL in NASH has been assessed using the disease-specific Chronic Liver Disease Questionnaire for NASH (CLDQ-NASH) and the generic EuroQol EQ-5D-5L. As the performance of these instruments relative to each other is unknown, we performed a cross-walk analysis of CLDQ-NASH to EQ-5D-5L using data from a real-world NASH population.

**Methods:**

Data were drawn from the Adelphi Real World 2019 NASH Disease Specific Programme, a cross-sectional survey of physicians and their patients in the United States. Patients with physician-diagnosed NASH completed a questionnaire that included the CLDQ-NASH and EQ-5D-5L. Mapping from CLDQ-NASH to EQ-5D-5L was done using tenfold cross-validation; performance was assessed using root-mean squared error as accuracy measure. Subgroup analyses compared performance of the models in obese versus non-obese patients and patients with versus without type 2 diabetes (T2D).

**Results:**

Data from 347 patients were included in this analysis. Overall, 2172 models were tested for predicting EQ-5D-5L index score from CLDQ-NASH score. The best model for this mapping was a generalized linear model using Gaussian distribution and a power link. The best model for mapping from CLDQ-NASH domains to the EQ-5D-5L was a fractional logistic model. Models performed better at predicting upper versus lower values of EQ-5D-5L, for non-obese versus obese patients, and for patients without versus with T2D.

**Conclusion:**

We describe a scoring algorithm for cross-walking the CLDQ-NASH to the EQ-5D-5L enabling health status comparisons of HRQoL across studies.

**Supplementary Information:**

The online version contains supplementary material available at 10.1186/s12955-023-02195-x.

## Plain English summary

Nonalcoholic steatohepatitis (NASH) is a progressive, life-threatening disease that can seriously affect patients’ lives if left untreated. Measuring how NASH and its treatments impact patients’ quality of life (QoL) is an important part of clinical trials. We do this by asking patients to fill in questionnaires, some that look at general aspects of QoL (generic instruments) and some that are specific to a disease or condition. Generic instruments are often used by regulators to decide whether new treatments offer value for money. Few studies have looked at QoL in NASH and little is known about how different questionnaires compare. We did this work to find a way of converting the results of a disease-specific questionnaire (the Chronic Liver Disease Questionnaire for NASH [CLDQ-NASH]) to a generic one (the EQ-5D-5L) that has been more widely studied and is often used by regulators to help in decision-making. We tested many models to find the best way of predicting EQ-5D-5L from CLDQ-NASH scores. Doing cross-walking like this allows comparisons to be made between studies using either of these questionnaires. This can then allow clinical trial data to be cross-walked and submitted for regulatory use.

## Introduction

Nonalcoholic steatohepatitis (NASH), a chronic, progressive disease characterized by fatty liver, liver cell injury, and inflammation, is a progression of nonalcoholic fatty liver disease, a spectrum of chronic liver disease characterized by excessive cytoplasmic retention of triglyceride [1]. There is a paucity of data regarding the prevalence of NASH in the general population [2]; however, studies have reported prevalence rates of 3–5% in the United States [3]. Prevalence rates are significantly increasing in the United States, up from 1.5% in 2010 to 2.8% in 2020 [4]. Individuals with NASH tend to have many comorbidities, and their management can place a considerable strain on public health services [5], particularly as there are currently no licensed NASH therapies [6].

The symptoms and management of NASH can impact the quality of life (QoL) of affected individuals and health-related quality of life (HRQoL) worsens as the disease progresses [7]. Assessment of HRQoL is important when evaluating treatments for individuals with NASH, for which a variety of patient-reported outcome measures (PROMs) have been developed. According to clinical experts, disease symptomatology in NASH is best assessed by disease-specific instruments such as the Chronic Liver Disease Questionnaire (CLDQ) [8]. The CLDQ and its NASH-specific version, the CLDQ-NASH, are among the most commonly used and validated instruments for assessing QoL in NASH clinical trials. CLDQ-NASH measures patient-reported outcomes (PROs) across six domains: abdominal symptoms, activity/energy, emotional health, fatigue, systemic symptoms, and worry [8]. Such disease-specific instruments enable in-depth evaluation of aspects of life most affected by a disease; however, results obtained using disease-specific instruments cannot easily be compared across trials because of differences in study designs, instruments used, and populations.

Cross-trial comparisons are best accomplished using generic preference-based instruments. Measuring HRQoL using generic instruments allows for comparison of outcomes across diseases and populations, and generic preference-based instruments can be used to generate utility values required in health economic evaluations [9]. The EuroQol EQ-5D-5L is a widely used generic instrument that measures outcomes in five dimensions: mobility, self-care, usual activities, pain/discomfort, and anxiety/depression [10]. Each dimension is rated using five severity levels: no problems, slight problems, moderate problems, severe problems, and extreme problems/unable to do. Responses are used to generate a single index score describing the patient’s health state. The EQ-5D was considered the most appropriate measure for this analysis as it is often used to measure health benefits in health technology assessments (HTAs) and economic modeling. Although the EQ-5D does not cover every symptom of every condition, its development process is such that it is the most widely used health utility measurement for a uniform approach across multiple diseases, and it is used in assessments conducted by the National Institute for Health and Care Excellence in the United Kingdom, which identified the EQ-5D 3-Levels questionnaire as its preferred instrument in 2008 and updated this to include the EQ-5D-5L in 2019 [11]. Similarly, use of generic preference-based measures is recommended by the Institute for Clinical and Economic Review in the United States, with the EQ-5D being the preferred instrument if available [12]. This provides consistency of decision-making with uniformity in health-state measurement, which enables trade-offs to be made concerning healthcare resource use prioritization between different unrelated health conditions.

In situations where generic instruments are not used in a clinical trial or other study, a process referred to as mapping or cross-walking can be used to predict utilities that might have been obtained [13]. The mapping process involves developing and validating usable algorithms that can map or link a disease-specific instrument such as the CLDQ to a generic preference-based instrument. Acceptable methodologies for rigorous mapping analysis have been outlined by organizations using results of these analyses for medical decision-making [13–15].

Although the CLDQ-NASH and the EQ-5D are used in HRQoL assessment of individuals with NASH [16, 17], their performance relative to each other is unknown. It is important to be able to compare results using these instruments across studies and assess equivalency of outcomes. Such comparisons are considered feasible as both contain measures on QoL – one a general measure of health status and one that is disease specific. Clearly the disease-specific measure will omit information that is considered by the general measure (better health for some patients, worse health for others for non-NASH reasons), but on average it may produce good estimates. A formal comparison, however, will allow us to know if using one of these instruments in a study is sufficient, the most likely scenario being use of the CLDQ-NASH in the absence of the EQ-5D-5L. We conducted a cross-walk analysis from the source measure, the CLDQ-NASH, to the target measure, the EQ-5D-5L, using patient-reported data from the Adelphi Real World NASH 2019 Disease Specific Programme™ (DSP), a large cross-sectional survey of physicians and their patients presenting in a real-world clinical setting.

## Methods

### Data source

This analysis used secondary data from the Adelphi Real World NASH DSP conducted in January to March 2019 in the United States. The DSP captured a combination of abstracted physician-reported medical record data and patient-reported survey data. The full methodology of the DSP has been published [18] and validated [19, 20]. Adelphi Real World standard operating procedures were followed regarding data quality and accuracy.

Physicians included in the DSP were hepatologists, gastroenterologists, endocrinologists, or primary care physicians (PCPs) who were personally responsible for managing and making treatment decisions for patients with NASH in the outpatient setting. Specialists were required to treat > 10 patients with NASH per month and PCPs were required to treat > 5 patients with NASH per month. Eligible physicians completed patient record forms for their next eight consulting patients with a physician-confirmed NASH diagnosis. Physicians reported information on demographics (including age, sex, ethnicity, and body mass index [BMI]) and clinical characteristics (including comorbidities and disease severity as stated by the treating physician). Obesity was defined as a current BMI ≥ 30 kg/m^2^. Physicians assigned patients a fibrosis score based on their own clinical judgment.

Patients included in the DSP were ≥ 18 years, with a physician-reported diagnosis of NASH, and were not involved in a NASH clinical trial at the time of data collection. To be representative of real-world clinical practice, a liver biopsy was not the sole requirement to confirm NASH diagnosis. Patients were invited to complete a voluntary questionnaire, which included the CLDQ-NASH and EQ-5D-5L.

All patients provided written informed consent for use of their anonymized and aggregated data for analysis and publication. Responses were anonymized to preserve respondent (physician and patient) confidentiality. Participating physicians and patients were assigned a study number to aid anonymous data collection and allow linkage of data during data collection and analysis. Data were de-identified and aggregated before receipt by Adelphi Real World. As this was an analysis of secondary data, specific institutional review board (IRB) approval was not required. However, the NASH DSP was submitted to the Western IRB (protocol number AG8065) in the United States and an IRB exemption granted. This review ensured the study was methodologically and ethically sound, validating that the study adhered to international ethical standards with no risk to participants.

### Outcome measures

#### CLDQ-NASH

The CLDQ-NASH includes 36 items, measured on a Likert scale from 1 to 7, with higher values representing better health. Each domain score is calculated as the mean of its constituent item scores; the total score is calculated as the mean of the six domain scores.

#### EQ-5D-5L

Responses to the five items in the EQ-5D-5L define a health state, for which an index score is generated to indicate its value relative to the general population. The EQ-5D-5L index score is calculated following the approach recommended by the EuroQol Group [21, 22]. Each patient is initially assigned a score of 1, with adjustments reducing the index score based on their responses to questions. These adjustments to EQ-5D-5L are made according to the US societal value set reported by Pickard et al. [21]. Index scores therefore range from 1.00 (full health) to -0.573 (lowest possible score). The EQ-5D-5L index score was not calculated for a patient if any data were missing.

Conceptual overlap between these two instruments was considered reasonable as similar concepts are evaluated [7].

### Statistical analysis

Our statistical analysis was conducted in line with current best practices for conducting mapping studies [13, 15] and this study used the MApping onto Preference-based measures reporting Standards reporting checklist [23].

All patients who fully completed the CLDQ-NASH and EQ-5D-5L questionnaires were included in the analysis.

Mapping from the CLDQ-NASH to the EQ-5D-5L was done using tenfold cross-validation, which uses the collected sample for training/estimation and validation [24]. In brief, patients in the dataset were randomly partitioned into ten subsamples. One subsample was retained as the validation dataset for testing the predictive model; the remaining nine subsamples were used as training data. The cross-validation process was repeated nine times, with each of the ten subsamples being used once as the validation dataset. Model accuracy measures were calculated on the combined validation data. These statistics may be considered “out of sample” as they were calculated on data not used in the predictive model. The collected sample was used for training and validation purposes.

The performance of each model was assessed in the tenfold cross-validation using root-mean squared error (RMSE) as accuracy measure. The RMSE is the square root of the variance of the residuals and can be interpreted as the standard deviation (SD) of the unexplained variance, or how concentrated the reported data are around a predicted line of best fit. Lower values of RMSE (lower spread) indicate better fit, and well-fitting models would ideally have an RMSE lower than the minimally important difference for the EQ-5D-5L (0.03–0.05 [25]). The RMSE was examined at the lower, middle, and upper parts of the predicted scale to identify if the model was a good fit for the full range of values. For the EQ-5D-5L, these parts were defined as the equally sized ranges of -0.573 to 0.049, -0.049 to 0.476, and 0.476 to 1, respectively.

The mean score was generated initially to provide a benchmark for other models. A variety of statistical models were generated: ordinary least squares linear regression, generalized linear models (GLMs) with gaussian inverse gaussian and gamma errors paired with identity, log and power links (powers ranged from -5 to 5 in increments of 0.1), fractional logit/probit, nonparametric local-linear regression, random forest, classification and regression tree, finite mixture models with a point mass at 1, and adjusted limited dependent variable mixture models. Some models constrained predictions to between 0 and 1, which necessitated rescaling the EQ-5D to these bounds, and rescaling the predictions. Only the CLDQ-NASH was used as independent variable(s). This was either the overall score or the six domain scores. Models were also assessed including splines of covariates (linear splines with 2–4 knots, and restricted cubic splines with 3–5 knots). Knots for linear splines were chosen so that they were equally spaced over the range of the covariate, and knots for restricted cubic splines were chosen following the recommendations of Harrell [26]. The model with the lowest RMSE in cross-validation was deemed the best.

Cross-walking between the CLDQ-NASH and EQ-5D-5L was conducted using the CLDQ-NASH total score and EQ-5D-5L index score. CLDQ-NASH domain scores were also mapped to the EQ-5D-5L index score.

Two subgroup analyses compared RMSEs for: (i) obese NASH (BMI ≥ 30 kg/m^2^) versus non-obese NASH (BMI < 30 kg/m^2^); and (ii) diabetic NASH (patients with NASH and type 2 diabetes [T2D]) versus nondiabetic NASH. Given the strong link between obesity and T2D [27–29], the high prevalence of obesity in people with diabetes (58% in National Health and Nutrition Examination Survey [NHANES] participants with diabetes from 1999 to 2020 [30]), and the increased risk of diabetes in the obese (43% in people with BMI ≥ 40 in NHANES participants from 1999 and 2006 [31]), we wished to independently evaluate the effects of NASH and remove the confounding effects of these conditions.

All analyses were conducted in Stata 17.0 (StataCorp LLC, College Station, TX, USA) [32].

## Results

### Participants

A total of 347 patients in the Adelphi Real World NASH DSP database completed a CLDQ-NASH, EQ-5D-5L, and with a corresponding physician-reported patient record form, and were eligible for inclusion in this analysis. Patient characteristics are shown in Table 1. Among the 347 patients, physicians stated that the fibrosis stage was F0 for 7.8% of patients, F1 for 9.6%, F2 for 16.7%, F3 for 16.4%, and F4 for 14.7%. Thus, 261 patients had a stated fibrosis stage. A fibrosis stage was not assigned by the physician for 86 patients (25%). Characteristics of patients with obesity/no obesity and T2D/non-T2D are also shown in Table 1. Tests performed are shown in Supplementary Table 1; pharmacological treatments prescribed for selected comorbidities are shown in Supplementary Table 2.

**Table 1:** Patient characteristics overall and according to obesity and type 2 diabetes status

	All patients (*n* = 347)	Obese (*n* = 249)	Non-obese (*n* = 98)	T2D (*n* = 194)	Non-T2D (*n* = 151)^a^
Mean age, years (SD)	55.6 (11.9)	55.2 (11.2)	56.9 (13.5)	57.5 (11.1)	53.3 (12.5)
Sex, n (%)					
Male	186 (53.6)	141 (56.6)	45 (45.9)	104 (53.6)	80 (53.0)
Female	161 (46.4)	108 (43.4)	53 (54.1)	90 (46.4)	71 (47.0)
Ethnicity, n (%)					
White/Caucasian	271 (78.1)	196 (78.7)	75 (76.5)	147 (75.8)	122 (80.8)
African American	28 (8.1)	23 (9.2)	5 (5.1)	18 (9.3)	10 (6.6)
Hispanic/Latino	18 (5.2)	13 (5.2)	5 (5.1)	14 (7.2)	4 (2.6)
Other	30 (8.6)	17 (6.8)	13 (13.3)	15 (7.7)	15 (9.9)
Mean BMI, kg/m^2^ (SD)	33.2 (5.8)	35.7 (4.8)	26.8 (2.7)	34.0 (6.1)	32.2 (5.3)
Comorbidities, n (%)^b^	(*n* = 345)	(*n* = 249)	(*n* = 96)	(*n* = 194)	(*n* = 151)
T2D	194 (56.2)	145 (58.2)	49 (51.0)	194 (100)	0
Hypertension	167 (48.4)	117 (47.0)	50 (52.1)	105 (54.1)	62 (41.1)
Myocardial infarction	20 (5.8)	13 (5.2)	7 (7.3)	15 (7.7)	5 (3.3)
Fibrosis stage, n (%)					
F0	27 (7.8)	18 (7.2)	9 (9.2)	16 (8.2)	11 (7.3)
F1	68 (19.6)	48 (19.3)	20 (20.4)	33 (17.0)	34 (22.5)
F2	58 (16.7)	45 (18.1)	13 (13.3)	32 (16.5)	26 (17.2)
F3	57 (16.4)	33 (13.3)	24 (24.5)	37 (19.1)	20 (13.2)
F4	51 (14.7)	36 (14.5)	15 (15.3)	29 (14.9)	22 (14.6)
Don’t know	86 (24.8)	69 (27.7)	17 (17.3)	47 (24.2)	38 (25.2)

*BMI* body mass index, *SD* standard deviation, *T2D* type 2 diabetes

^a^Don’t know: *n* = 2

^b^Occurring in > 5% of patients. Patients could have ≥ 1 comorbidity

### CLDQ-NASH and EQ-5D-5L scores

**Fig. 1:**
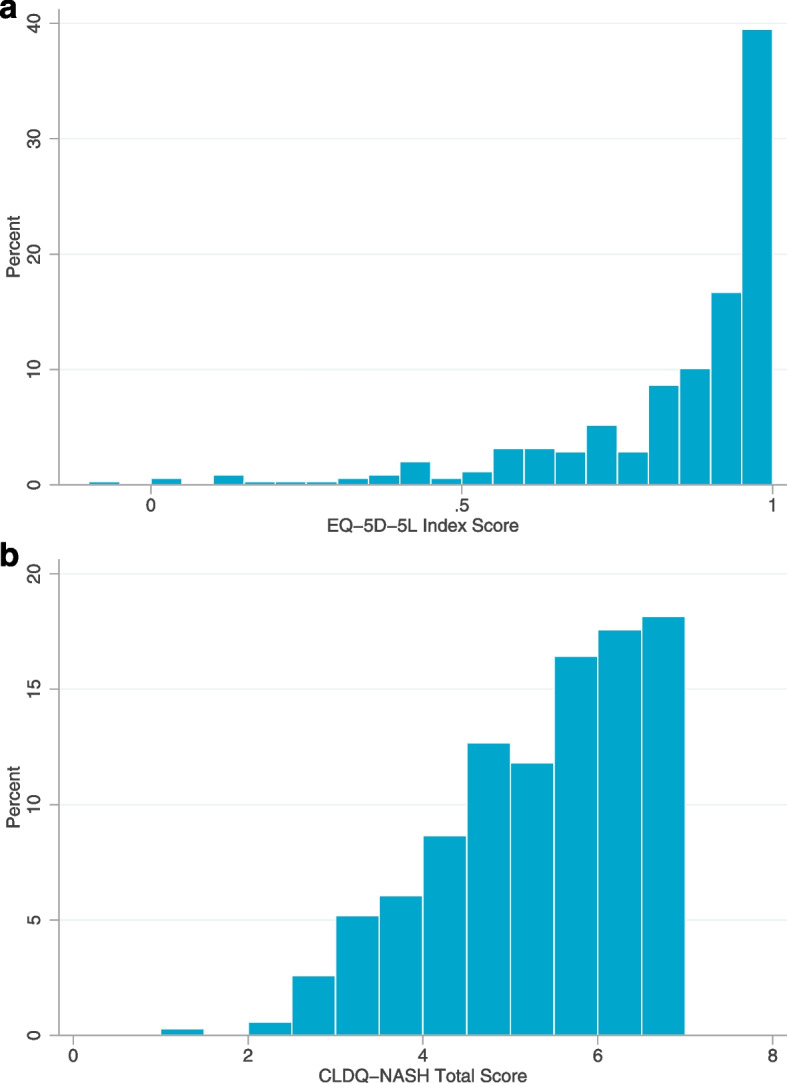
Histograms showing distribution of (**a**) EQ-5D-5L index and (**b**) CLDQ-NASH total scores. *CLDQ-NASH* Chronic Liver Disease Questionnaire – Nonalcoholic Steatohepatitis

The distribution of CLDQ-NASH and EQ-5D-5L scores is shown in Fig. 1 and descriptive details are given in Table 2. As expected, many patients in the overall sample (*n* = 137; 39%) had an EQ-5D-5L index score of 1.00. When the stratification factor of obesity was considered, the mean index score for obese patients was 0.840 (SD 0.210), with 44% scoring 1.00; the mean index score for non-obese patients was 0.891 (SD 0.157), with 38% scoring 1.00 (Supplementary Fig. 1). Patients with and without T2D had EQ-5D-5L index scores of 0.836 (SD 0.214; 36% scoring 1) and 0.875 (SD 0.174; 43% scoring 1), respectively. The overall population norm for the United States is 0.851 [SD0.205]) [33].

**Table 2:** Summary of CLDQ-NASH and EQ-5D-5L data in patients with NASH

	Overall (*n* = 347)	Obese (*n *= 249)	Non-obese (*n* = 98)	T2D (*n *= 194)	Non-T2D (*n* = 151)
**CLDQ-NASH**					
*Overall*					
Mean (SD)	5.4 (1.2)	5.3 (1.2)	5.6 (1.1)	5.2 (1.1)	5.5 (1.2)
Median (IQR)	5.6 (4.6, 6.3)	5.4 (4.5, 6.2)	5.7 (5.0, 6.4)	5.4 (4.5, 6.0)	5.8 (4.7, 6.6)
*Abdominal*					
Mean (SD)	5.3 (1.4)	5.2 (1.4)	5.5 (1.3)	5.2 (1.4)	5.4 (1.5)
Median (IQR)	5.7 (4.3, 6.7)	5.3 (4.0, 6.3)	5.7 (4.3, 6.7)	5.3 (4.0, 6.3)	5.7 (4.3, 7.0)
*Activity*					
Mean (SD)	5.3 (1.3)	5.2 (1.3)	5.5 (1.2)	5.1 (1.3)	5.6 (1.3)
Median (IQR)	5.4 (4.4, 6.4)	5.2 (4.2, 6.2)	5.6 (4.8, 6.6)	5.2 (4.2, 6.0)	5.8 (4.8, 6.8)
*Emotion*					
Mean (SD)	5.4 (1.3)	5.3 (1.3)	5.6 (1.3)	5.3 (1.3)	5.6 (1.3)
Median (IQR)	5.6 (4.4, 6.6)	5.6 (4.4, 6.4)	5.8 (4.6, 6.7)	5.4 (4.4, 6.3)	5.9 (4.8, 6.8)
*Fatigue*					
Mean (SD)	5.0 (1.4)	4.9 (1.4)	5.3 (1.3)	4.8 (1.3)	5.3 (1.4)
Median	5.2 (4.0, 6.2)	5.2 (3.8, 6.0)	5.3 (4.7, 6.2)	5.2 (3.7, 5.8)	5.3 (4.3, 6.5)
*Systemic*					
Mean (SD)	5.7 (1.1)	5.6 (1.1)	5.9 (1.1)	5.5 (1.1)	5.8 (1.2)
Median (IQR)	6.0 (4.8, 6.7)	5.8 (4.7, 6.5)	6.2 (5.3, 6.7)	5.8 (4.7, 6.3)	6.2 (5.0, 6.8)
*Worry*					
Mean (SD)	5.4 (1.3)	5.4 (1.3)	5.6 (1.3)	5.3 (1.3)	5.5 (1.4)
Median (IQR)	5.7 (4.6, 6.6)	5.7 (4.6, 6.4)	6.0 (4.7, 6.6)	5.7 (4.4, 6.4)	5.9 (4.7, 6.7)
**EQ-5D-5L – US**					
Mean (SD)	0.854 (0.198)	0.840 (0.210)	0.891 (0.157)	0.836 (0.214)	0.875 (0.174)
Median (IQR)	0.932 (0.787, 1.000)	0.904 (0.750, 1.000)	0.940 (0.845, 1.000)	0.904 (0.723, 1.000)	0.940 (0.817, 1.000)

*CLDQ-NASH* Chronic Liver Disease Questionnaire – Nonalcoholic Steatohepatitis, *IQR* interquartile range, *NASH* nonalcoholic steatohepatitis, *SD* standard deviation, *US* United States

A similar pattern was observed for CLDQ-NASH scores. Mean scores for obese and non-obese patients were 5.3 (SD 1.2) and 5.6 (SD 1.1), respectively. Score distribution according to T2D status is shown in Supplementary Fig. 2. A similar pattern was observed when T2DM was considered, with mean scores for patients with and without T2D of 5.2 (SD 1.1) and 5.5 (SD 1.2), respectively.

### Mapping analysis

Overall, 2172 models were tested for predicting the EQ-5D-5L index score from the CLDQ-NASH score. The key findings of this analysis are summarized in Table 3. Best models were defined as those with the lowest overall RMSE.

**Table 3:** Best fitting models for predicting EQ-5D-5L from CLDQ-NASH and the cross-validation results

Model	Description	Statistical model	Overall RMSE	Lower RMSE	Middle RMSE	Upper RMSE	Obese RMSE	Non-obese RMSE	T2D RMSE	Non-T2D RMSE
Mean	Benchmark	Constant model	0.1980	0.9164	0.5756	0.1372	0.2111	0.1602	0.2143	0.1756
GLM family (Gaussian)	Best model using overall CLDQ-NASH score as independent variables	GLM with gaussian family and power link (power -0.3), cubic splines with 3 knots	0.1455	0.7644	0.3517	0.1146	0.1505	0.1318	0.1583	0.1249
Fractional logistic	Best model using the CLDQ-NASH domains as independent variables	Fractional logistic, cubic splines with 3 knots	0.1452	0.7698	0.3477	0.1149	0.1533	0.1221	0.1585	0.1242

*CLDQ-NASH* Chronic Liver Disease Questionnaire – Nonalcoholic Steatohepatitis, *GLM* generalized linear model, *RMSE* root-mean squared error, *T2D* type 2 diabetes

**Fig. 2:**
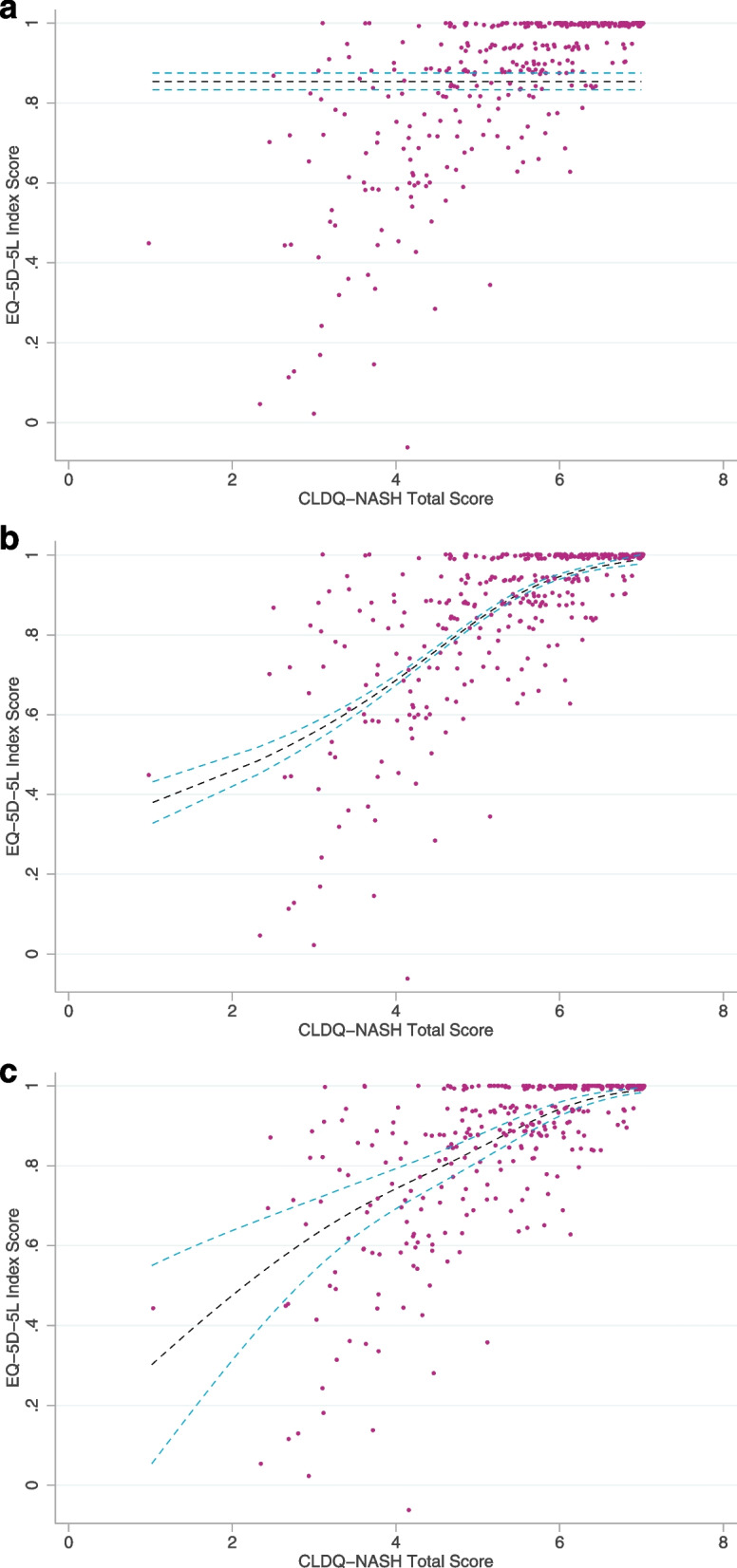
Models for predicting EQ-5D-5L from CLDQ-NASH. Patient-recorded or actual values are shown in pink, the black dashed line represents the CLDQ-NASH derived values, and the blue dashed lines represent 95% confidence intervals of the predicted values. **a** Mean model. **b** Generalized linear model with Gaussian family and power link (power -0.3), with cubic splines, 3 knots) using total score as independent variable; (**c**) fractional logistic model, with cubic splines, 3 knots and using domain scores as independent variables. **C** assumes all subdomains rise at the same rate to create the total score. *CLDQ-NASH* Chronic Liver Disease Questionnaire – Nonalcoholic Steatohepatitis

The mean model predicted an EQ-5D-5L index score of 0.854 for all patients, the benchmark for other models (Fig. 2a). The best model for the CLDQ-NASH overall score was the GLM with gaussian family and power link (power -0.3), using cubic splines with three knots (Fig. 2b), which had an overall RMSE of 0.1455. Fitted values for the CLDQ-NASH were within the range of values possible for the EQ-5D. The best model for the CLDQ-NASH domains was the fractional logistic model, using cubic splines with three knots (Fig. 2c), which had an overall RMSE of 0.1452. Mapping of individual domains is shown in Supplementary Fig. 3. It should be noted that Supplementary Fig. 3 is artificial in that the predicted EQ-5D values are shown assuming that all other domains are held at their average values as one domain changes; in patients, it is unlikely that one domain would increase without an associated increase seen in any other domain.

The best model using the CLDQ-NASH domains is associated with a lower RMSE (0.1452) than the best model using the CLDQ-NASH overall score (RMSE 0.1455), hence this was the preferred model.

As shown in Table 3, models performed better at predicting upper values (RMSE 0.1146) of EQ-5D-5L than at lower values (RMSE 0.7644). For the subgroup analyses, the model performed better for non-obese patients (RMSE 0.1318) than for obese patients (RMSE 0.1505), and for patients without T2D (RMSE 0.1249) than for those with T2D (RMSE 0.1583).

Detailed instructions for application of the mapping results to generate EQ-5D-5L utility scores are shown in Supplementary Tables 3 and 4.

## Discussion

The types of PROMs used in clinical trials and those required for cost-effectiveness analysis often differ. Generic preference-based instruments, such as the EQ-5D, are widely used in economic evaluations required as part of HTAs but are often not used in the clinical trial setting as regulatory bodies frequently require the use of disease-specific instruments [12, 34–36]. Mapping provides a means of generating reliable health state utility values when no preference-based measure is included in the study. This requires a degree of overlap between the descriptive systems of both instruments and administration of both on the same population. HRQoL assessment in patients with NASH is a relatively underdeveloped area of research. The CLDQ-NASH was validated for use in NASH in 2019 and is less widely used than other HRQoL instruments. Early stages of NASH are frequently asymptomatic, at which point a symptom-focused questionnaire may be less informative than a generic instrument such as the EQ-5D-5L. To our knowledge, therefore, no direct comparison between CLDQ-NASH and EQ-5D-5L is available to facilitate HTA submissions.

We conducted a cross-mapping analysis for the CLDQ-NASH as source measure to the EQ-5D-5L as target measure in a real-world population of patients with NASH in the United States and identified models best suited to predict EQ-5D-5L scores considering overall and domain scores of the CLDQ-NASH. The best model was the GLM family (Gaussian) link (power -0.3) using cubic splines with three knots, which produced fitted values for the CLDQ-NASH within the range of values possible for the EQ-5D-5L. The best model for the CLDQ-NASH domains – the fractional logistic model with cubic splines and three knots – slightly outperformed the model in which only the total score was used. It was also the best-performing model for predicting higher EQ-5D-5L values. In situations where information is not provided for domains, however, the model using the total score may be used. Given that the RMSE values are relatively similar for both models, this should not correspond to a great loss in predictive power.

Approximately 40% of patients reported an EQ-5D-5L index score of 1 (full health), and approximately 4% of patients had a CLDQ-NASH total score of 7 (best score possible). An EQ-5D score of 1.00 is not unusual and in many diseases a significant portion of patients will have a score of 1 [37–39]. This type of ceiling effect can be problematic in models, but both the GLM and fractional logistic cubic spline predicted EQ-5D values of approximately 0.3 − 0.4 for the lower end of the CLDQ-NASH total score, and a value close to 1 at the upper end. A cursory examination of Fig. 2a, b, and c shows there is clear value in using the CLDQ-NASH to predict EQ-5D as lines of best fit appear to intuitively fit the data. Some differences were seen in the shape of the line of best fit, but predicted values were similar throughout, which gave rise to similar RMSE values, as shown in Table 3.

Both models performed better in non-obese versus obese patients, and in patients who did not have T2D versus those who did. This is likely a result of obese patients and those with T2D having greater variability in EQ-5D-5L values because of the range of complications that frequently exist in individuals with these conditions [40, 41], and the coexistence of obesity and T2D in many patients [42]. We observed that the two best models performed less well at predicting lower versus upper values of EQ-5D-5L. As HRQoL declines in patients with multiple comorbidities [7], it is expected that the models would work less well in these patients. Ultimately, HRQoL in patients with multiple comorbidities and NASH is likely to be affected by several factors, with the result that changes in NASH may be difficult to identify if the only PROM is a generic one, whereas disease-specific measures may be better able to detect these changes.

Few studies have reported CLDQ-NASH and EQ-5D scores for patients with NASH. Our data are in line with those that did, including the study by Younossi et al., in which EQ-5D scores of 0.812 and 0.831 were reported for patients with liver stiffness values by transient elastography of ≥ 11.4 and < 11.4 kPa, respectively, and 0.821 and 0.844 for those with and without T2D, respectively [43]. The same study reported CLDQ-NASH scores of 5.1 and 5.3 for patients with and without T2D, respectively, similar to the values reported in our study. O’Hara et al. reported somewhat lower overall EQ-5D (0.75) and CLDQ-NASH scores (4.4) for patients with NASH in the United States in the multinational GAIN study than reported in this analysis (0.85 and 5.4, respectively) [44].

HRQoL score in patients with NASH has also been reported to correlate with obesity and T2D. Huber et al. observed significantly lower CLDQ overall scores in patients with BMI > 30 kg/m^2^ versus those with lower BMI (4.8 vs 5.5, respectively; *p* < 0.001) [45]. In contrast, David et al. reported that, although patients with severe obesity (BMI > 40 kg/m^2^) had significantly lower HRQoL than those who did not, there was no independent relationship between BMI and Medical Outcomes Study Short-Form (36-item) Health Survey (SF-36) score after adjustment for other covariates [46]. In their analysis of patients with NASH in Europe, Balp et al. compared SF-36 scores in the general population, patients with NASH, and patients with T2D [47]. Although patients with NASH had significantly worse HRQoL than the general population, patients with NASH did not differ from those with T2D in their SF-36 Physical Component Scores, despite having worse mental component scores and greater healthcare utilization.

Some limitations of this analysis warrant consideration. Our mapping algorithms did not perform as well at lower EQ-5D values, an expected consequence of the EQ-5D curve that has been described in other studies [9, 38] and confounded in part by the fact that our patients were undergoing treatment in the outpatient setting, with the result that capture of those with early-stage liver disease and decompensating cirrhosis was limited. We present the case for predicting EQ-5D using CLDQ-NASH, assuming that there is a degree of overlap in the descriptive systems of both instruments. It is also a fair assumption that there is some degree of non-overlap, and it may be this that drives variation around the predictions of EQ-5D, i.e. CLDQ-NASH does not measure all the facets of a patient’s life that are covered by the EQ-5D. Models would likely be improved by also utilising covariates for comorbid conditions and their associated severity; this, however, this was beyond the scope of the present study, the purpose of which was to provide a mapping algorithm directly from CLDQ-NASH to EQ-5D. Future studies could explore the domain overlap of PROs and the interplay of comorbid conditions on these PRO domains. The population size meant that a relatively small number of patients with advanced fibrosis (as classified by the physician) were included. This impacted on the number of patients with low EQ-5D scores and resulted in fewer observations upon which to base mapping. Analysis was restricted to patients who were willing to participate in completing questionnaires, a general limitation with all real-world research. As each patient completed both instruments, however, the study is internally consistent. This analysis only included patients from the United States. If the results were to be applied to other populations, some caution might be needed with interpretation taking into consideration that a patient recruited from outside the United States might score one of the instruments differently. In addition, different countries assign slightly different value sets to the EQ-5D, such that scores may not be the same even when responses are identical [48]. Finally, consistent with all mapping analysis, modeling results in inherent information loss that creates uncertainty when compared with direct EQ-5D-5L measurement [49]. However, mapping is often the only feasible way to conduct cost-utility analysis when direct evidence is unavailable. One key strength of the study was that patients were recruited by their treating specialist and only those with a physician-confirmed diagnosis of NASH were included. Additionally, this is the first known study to provide a NASH mapping algorithm from a commonly used disease-specific instrument to the EQ-5D-5L specifically with the intention of inclusion in an economic evaluation. Notably, detailed instructions have been provided to allow readers to generate utility scores from their own data.

## Conclusion

In conclusion, this study provides a scoring algorithm for cross-walking the CLDQ-NASH with the EQ-5D-5L that might be used in economic evaluations, allowing for comparisons across studies in which either one of these instruments is used. The RMSE values described herein provide evidence that the relationship between the two measures is sufficiently strong that a patient’s EQ-5D index may be successfully predicted from their CLDQ-NASH score. As studies may be conducted using either measure, results may be considered together, allowing stronger conclusions to be drawn. Further research is needed to confirm these findings.

### Supplementary Information

**Additional file 1: Supplementary Table 1.** Tests performed. **Supplementary Table 2.** Treatments received for select comorbidities. **Supplementary Table 3.** Best model for total score and its application: GLM family (gaussian) link(power -0.3) (cubic splines, 3 knots). **Supplementary Table 4.** Best model with domains and its application: Fractional logistic (cubic splines, 3 knots). **Supplementary Fig. 1.** Distribution of: (a) EQ-5D-5L index and (b) CLDQ-NASH total scores in patients considered obese and not obese. CLDQ-NASH Chronic Liver Disease Questionnaire – Nonalcoholic Steatohepatitis. **Supplementary Fig. 2.** Distribution of: (a) EQ-5D-5L index and (b) CLDQ-NASH total scores in patients with and without T2D. CLDQ-NASH Chronic Liver Disease Questionnaire – Nonalcoholic Steatohepatitis; T2D type 2 diabetes. **Supplementary Fig. 3.** Predicting EQ-5D-5L from CLDQ-NASH domains: fractional logistic model (with cubic splines, 3 knots). Patient-recorded or actual values are shown in pink, the black dashed line represents the CLDQ-NASH derived values, and the blue dashed lines represent 95% confidence intervals of the predicted values. CLDQ-NASH Chronic Liver Disease Questionnaire – Nonalcoholic Steatohepatitis.

## Data Availability

All data generated or analyzed during this study are included in this article and its supplementary information files.

## Abbreviations

BMI: Body mass index
CLDQ: Chronic Liver Disease Questionnaire
DSP: Disease Specific Programme™
GLM: Generalized linear model
HRQoL: Health-related quality of life
HTA: Health technology assessment
IRB: Institutional review board
IQR: Interquartile range
NASH: Nonalcoholic steatohepatitis
NHANES: National health and nutrition examination survey
PCP: Primary care physician
PROM: Patient-reported outcome measure
PRO: Patient-reported outcome
QoL: Quality of life
RMSE: Root-mean squared error
SD: Standard deviation
T2D: Type 2 diabetes
US: United States
