# Supplementary material for: Cross-walk of the Chronic Liver Disease Questionnaire for Nonalcoholic Steatohepatitis (CLDQ-NASH) and the EuroQol EQ-5D-5L in patients with NASH
Source: Health Qual Life Outcomes. 2023 Oct 14;21:113. doi: 10.1186/s12955-023-02195-x (PMC10576276; doi:10.1186/s12955-023-02195-x)

# Cross-walk of the Chronic Liver Disease Questionnaire for Nonalcoholic Steatohepatitis (CLDQ-NASH) and the EuroQol EQ-5D-5L in patients with NASH

Jesse Fishman^1*^, Victoria Higgins^2^, James Piercy^2^, James Pike^2^

^1^Madrigal Pharmaceuticals, Conshohocken, PA, USA; ^2^Adelphi Real World, Bollington, UK

*Correspondence: Jesse Fishman jfishman@madrigalpharma.com

# Supplementary information

**Supplementary Table 1** Tests performed

| **Most recent test** | **Value** |
| --- | --- |
| HbA_1c_ (%) |  |
| n | 136 |
| Mean | 6.9 |
| SD | 1.2 |
| Median | 6.9 |
| IQR | 6.0–7.9 |
| Min, Max | 3.0, 11.0 |
| ALT (U/L) |  |
| n | 238 |
| Mean | 65.4 |
| SD | 36.7 |
| Median | 60.0 |
| IQR | 38.0–85.0 |
| Min, Max | 1.0, 221.0 |
| AST (U/L) |  |
| n | 241 |
| Mean | 66.8 |
| SD | 143.8 |
| Median | 51.0 |
| IQR | 36.0–76.0 |
| Min, Max | 4.0, 2228.9 |
| GGT (U/L) |  |
| n | 40 |
| Mean | 55.6 |
| SD | 24.6 |
| Median | 52.0 |
| IQR | 39.5–67.0 |
| Min, Max | 0.9, 145.0 |
| ALP (U/L) |  |
| n | 140 |
| Mean | 107.8 |
| SD | 42.7 |
| Median | 103.0 |
| IQR | 78.0–137.5 |
| Min, Max | 29.0, 242.0 |
| Total bilirubin (mg/dL) |  |
| n | 165 |
| Mean | 1.3 |
| SD | 0.7 |
| Median | 1.1 |
| IQR | 0.7–1.8 |
| Min, Max | 0.2, 3.0 |
| Platelet count (10^9^/L) |  |
| n | 139 |
| Mean | 172.7 |
| SD | 103.5 |
| Median | 163.0 |
| IQR | 94.0–235.0 |
| Min, Max | 14.0, 400.0 |
| Serum albumin (g/L) |  |
| n | 134 |
| Mean | 35.9 |
| SD | 10.0 |
| Median | 38.0 |
| IQR | 29.0–43.0 |
| Min, Max | 10.0, 62.0 |
| INR (g/L) |  |
| n | 84 |
| Mean | 1.3 |
| SD | 0.4 |
| Median | 1.2 |
| IQR | 1.0–1.4 |
| Min, Max | 0.6, 2.6 |
| ELF |  |
| n | 4 |
| Mean | 2.3 |
| SD | 0.5 |
| Median | 2.0 |
| IQR | 2.0–2.5 |
| Min, Max | 2.0, 3.0 |
| MELD |  |
| n | 21 |
| Mean | 10.9 |
| SD | 5.6 |
| Median | 9.0 |
| IQR | 6.0–15.0 |
| Min, Max | 2.0, 25.0 |
| NAFLD fibrosis score |  |
| n | 24 |
| Mean | 1.8 |
| SD | 1.3 |
| Median | 2.0 |
| IQR | 0.9–3.0 |
| Min, Max | -1.6, 3.0 |
| FIB-4 |  |
| n | 14 |
| Mean | 1.9 |
| SD | 0.5 |
| Median | 2.0 |
| IQR | 1.8–2.0 |
| Min, Max | 1.0, 3.1 |

*ALP* alkaline phosphatase; *AST* aspartate transaminase; *ALT* alanine transaminase; *BMI* body mass index; *ELF* enhanced liver fibrosis; *FIB-4* Fibrosis-4 Index; *GGT* γ-glutamyltransferase; *HbA_1c_* glycated hemoglobin; *INR* international normalized ratio; *IQR* interquartile range; *MELD* Model for End-Stage Liver Disease; *Max* maximum*; Min* minimum*; NAFLD* nonalcoholic fatty liver disease; *SD* standard deviation.

**Supplementary Table 2** Treatments received for select comorbidities

| **Current treatment, n (%)** | **Patients receiving treatment (n=347)** |
| --- | --- |
| Statin | 197 (56.8) |
| GLP-1 receptor agonist | 57 (16.4) |
| SGLT2 inhibitor | 17 (4.9) |

*GLP-1* glucagon-like peptide-1; *SGLT2* sodium glucose co-transporter-2.

**Supplementary Table 3** Best model for total score and its application: GLM family(gaussian) link(power -0.3) (cubic splines, 3 knots)

Generalized linear models: Number of observations = 347

Variance function: V(u) = 1 [Gaussian]

Link function: g(u) = u^(-0.3) [Power]

| **EQ-5D_US** | **Coefficient** | **SE** | **z** | **P>\|z\|** | **95% CI** |
| --- | --- | --- | --- | --- | --- |
| CLDQ_TOTAL_CUB31 | -0.0732146 | 0.0072839 | -10.05 | 0.000 | -0.0874907 to -0.0589385 |
| CLDQ_TOTAL_CUB32 | 0.034334 | 0.007069 | 4.86 | 0.000 | 0.0204791−0.048189 |
| Constant term | 1.412118 | 0.0338873 | 41.67 | 0.000 | 1.3457−1.478536 |

*CI* confidence interval; *SE* standard error.

**Supplementary Table 4** Best model with domains and its application: Fractional logistic (cubic splines, 3 knots)

Fractional logistic regression: Number of observations = 347

| **EQ-5D_US** | **Coefficient** | **SE** | **z** | **P>\|z\|** | **95% CI** |
| --- | --- | --- | --- | --- | --- |
| CLDQ_AB_CUB31 | -0.0203931 | 0.1062948 | -0.19 | 0.848 | -0.2287272−0.187941 |
| CLDQ_AB_CUB32 | 0.1930467 | 0.1518087 | 1.27 | 0.203 | -0.1044929−0.4905863 |
| CLDQ_AC_CUB31 | 0.0806537 | 0.1232006 | 0.65 | 0.513 | -0.160815−0.3221224 |
| CLDQ_AC_CUB32 | 0.4729996 | 0.2084348 | 2.27 | 0.023 | 0.0644748−0.8815244 |
| CLDQ_EM_CUB31 | 0.0460203 | 0.1479132 | 0.31 | 0.756 | -0.2438843−0.3359249 |
| CLDQ_EM_CUB32 | 0.0290114 | 0.2103674 | 0.14 | 0.890 | -0.3833012−0.441324 |
| CLDQ_FA_CUB31 | 0.4342395 | 0.1366006 | 3.18 | 0.001 | 0.1665072−0.7019717 |
| CLDQ_FA_CUB32 | -0.2892396 | 0.2035091 | -1.42 | 0.155 | -0.68811−0.1096308 |
| CLDQ_SY_CUB31 | -0.1537929 | 0.123513 | -1.25 | 0.213 | -0.395874−0.0882882 |
| CLDQ_SY_CUB32 | 0.4095309 | 0.2255648 | 1.82 | 0.069 | -0.032568−0.8516297 |
| CLDQ_WO_CUB31 | 0.0868236 | 0.1306953 | 0.66 | 0.506 | -0.1693344−0.3429817 |
| CLDQ_WO_CUB32 | -0.0955588 | 0.1664001 | -0.57 | 0.566 | -0.421697−0.2305794 |
| Constant term | -0.254815 | 0.4228632 | -0.60 | 0.547 | -1.083612−0.5739818 |

*CI* confidence interval; *SE* standard error.

**Instructions for reproducing/using the analysis and applying to another dataset:**

1. CLDQ-NASH Total Score

Create two variables representing the restricted cubic spline of the CLDQ-NASH total score. Knots are placed at 3.627963, 5.576984, and 6.837989. In the regression above, these variables are named CLDQ_TOTAL_CUB31 (original linear variable) and CLDQ_TOTAL_CUB32 (non-zero values begin when CLDQ_TOTAL_CUB31 is 3.627963).

EQ-5D predictions are then given by the formula:

EQ-5D=(-0.0732146*CLDQ_TOTAL_CUB31 + 0.034334*CLDQ_TOTAL_CUB32 + 1.412118)^-(1/0.3)

1. CLDQ domains

Create two variables representing the restricted cubic spline of each CLDQ-NASH domain score:

CLDQ-NASH Abdominal - knots @ 3.333333, 5.666667, 7. Variables named CLDQ_AB_CUB31 and CLDQ_AB_CUB32.

CLDQ-NASH Activity - knots @ 3.4, 5.4, 7. Variables named CLDQ_AC_CUB31 and CLDQ_AC_CUB32.

CLDQ-NASH Emotion - knots @ 3.555556, 5.555556, 7. Variables named CLDQ_EM_CUB31 and CLDQ_EM_CUB32.

CLDQ-NASH Fatigue - knots @ 3, 5.166667, 6.866667. Variables named CLDQ_FA_CUB31 and CLDQ_FA_CUB32.

CLDQ-NASH Systemic - knots @ 4, 6, 7. Variables named CLDQ_SY_CUB31 and CLDQ_SY_CUB32.

CLDQ-NASH Worry - knots @ 3.428571, 5.714286, 7. Variables named CLDQ_WO_CUB31 and CLDQ_WO_CUB32.

EQ-5D predictions are then given by the formula:

EQ-5D = 1/(1+exp(-F))

Where F = -0.0203931*CLDQ_AB_CUB31 + 0.1930467*CLDQ_AB_CUB32 + 0.0806537*CLDQ_AC_CUB31 + 0.4729996*CLDQ_AC_CUB32 + 0.0460203*CLDQ_EM_CUB31 + 0.0290114*CLDQ_EM_CUB32 + 0.4342395*CLDQ_FA_CUB31 - 0.2892396*CLDQ_FA_CUB32 - 0.1537929*CLDQ_SY_CUB31 + 0.4095309*CLDQ_SY_CUB32 + 0.0868236*CLDQ_WO_CUB31 - 0.0955588*CLDQ_WO_CUB32 -0.254815

**Supplementary Fig. 1** Distribution of: (a) EQ-5D-5L index and (b) CLDQ-NASH total scores in patients considered obese and not obese. *CLDQ-NASH* Chronic Liver Disease Questionnaire – Nonalcoholic Steatohepatitis.

a


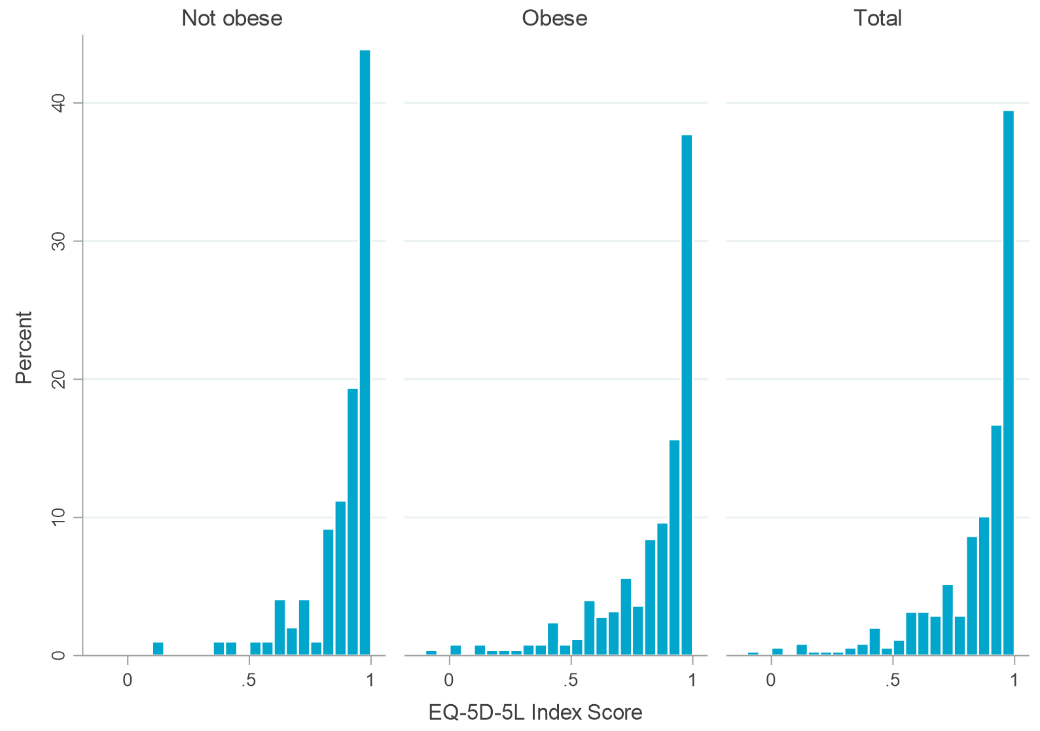


b


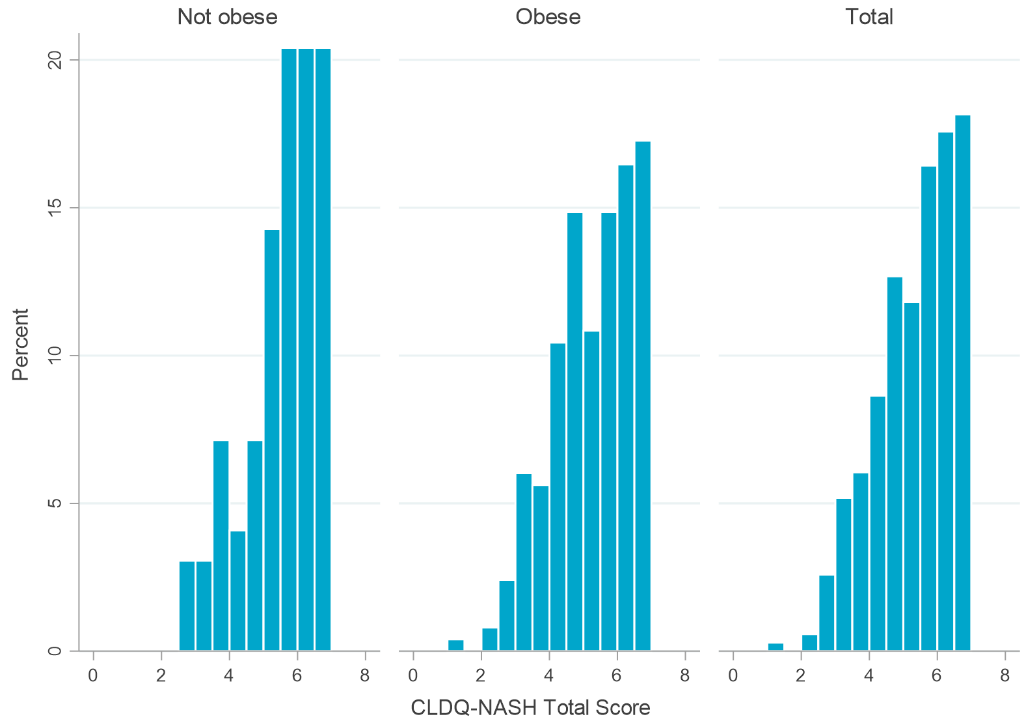


**Supplementary Fig. 2** Distribution of: (a) EQ-5D-5L index and (b) CLDQ-NASH total scores in patients with and without T2D. *CLDQ-NASH* Chronic Liver Disease Questionnaire – Nonalcoholic Steatohepatitis; *T2D* type 2 diabetes.

a


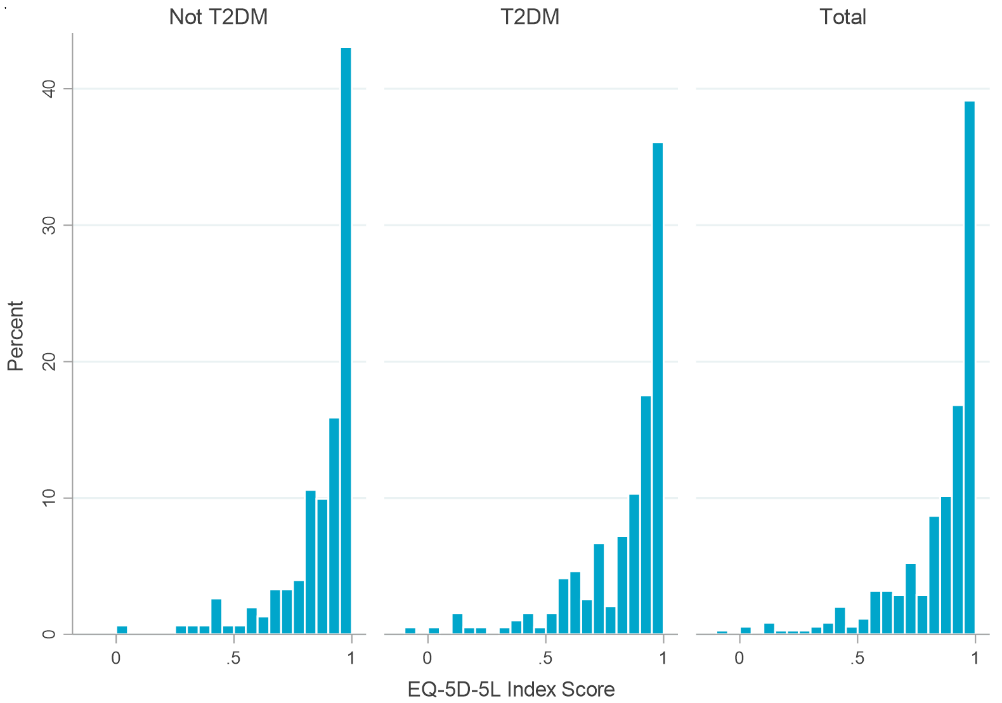


b


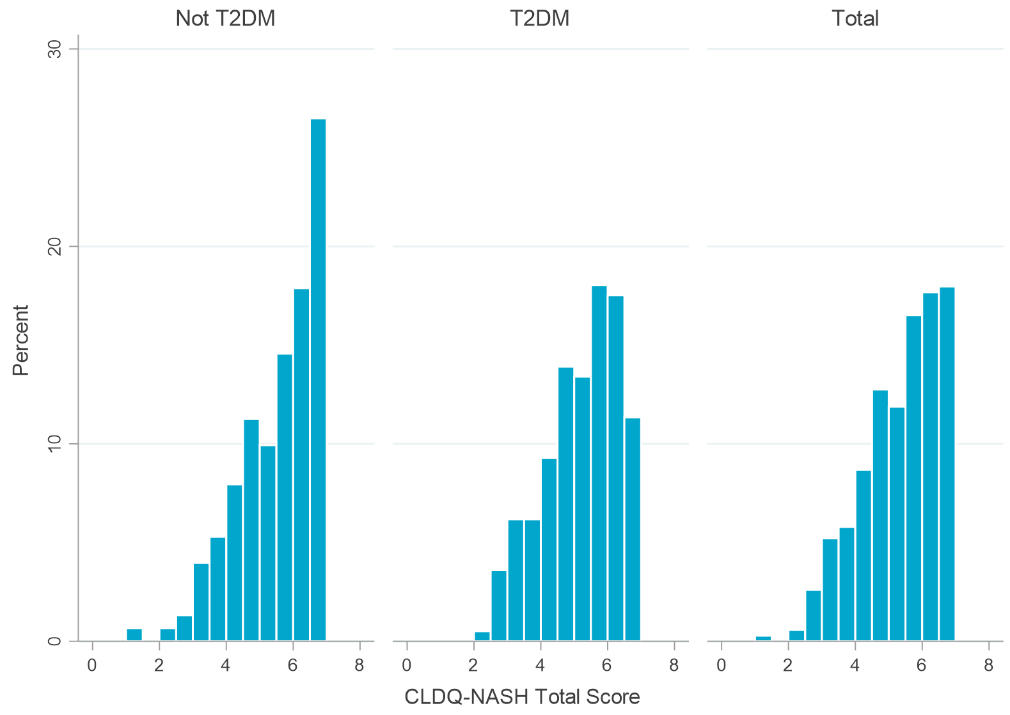


**Supplementary Fig. 3** Predicting EQ-5D-5L from CLDQ-NASH domains: fractional logistic model (with cubic splines, 3 knots). Patient-recorded or actual values are shown in pink, the black dashed line represents the CLDQ-NASH derived values, and the blue dashed lines represent 95% confidence intervals of the predicted values. *CLDQ-NASH* Chronic Liver Disease Questionnaire – Nonalcoholic Steatohepatitis.


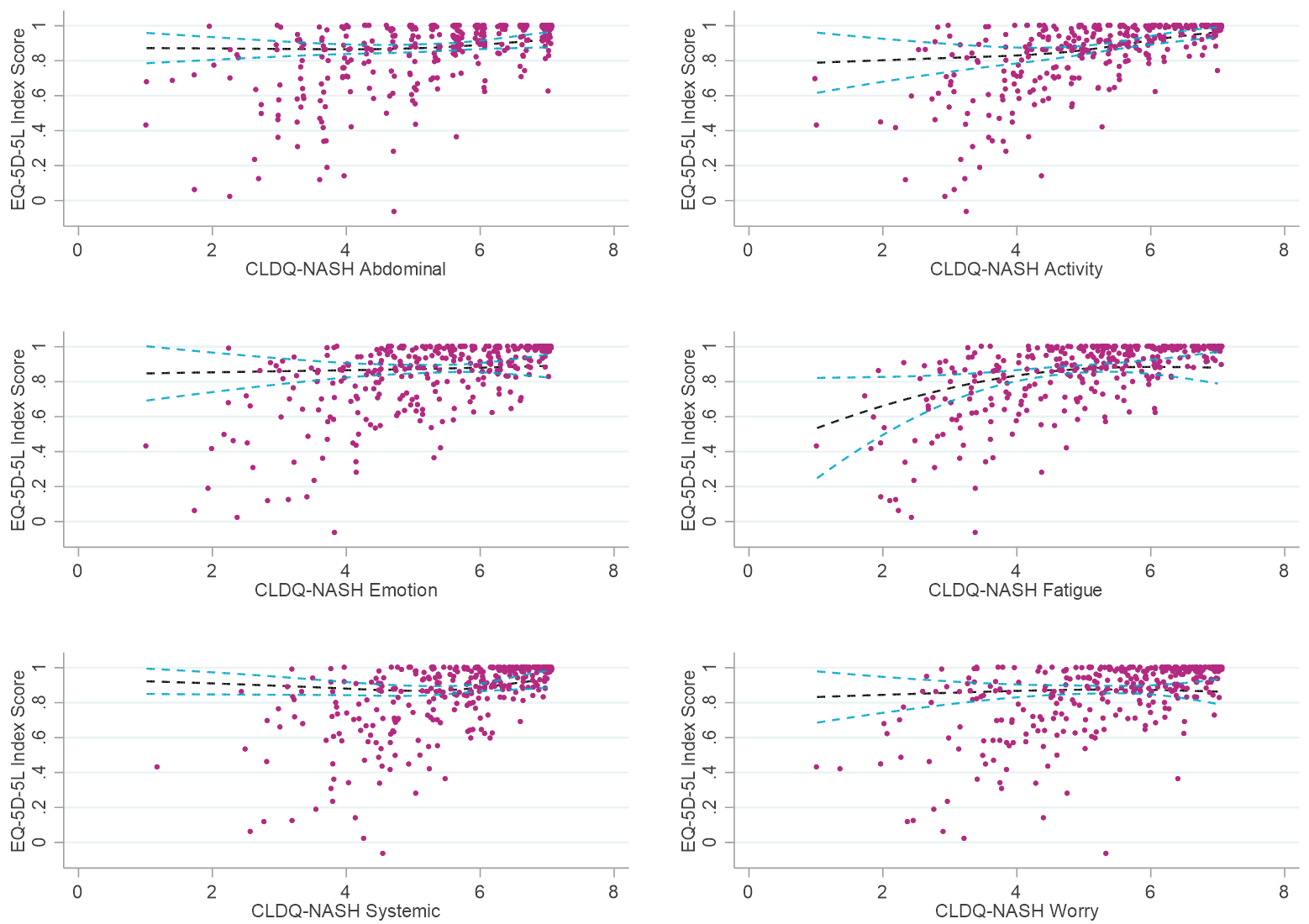

Supplement: Supplementary file 1 — Additional file 1: Supplementary Table 1. Tests performed. Supplementary Table 2. Treatments received for select comorbidities. Supplementary Table 3. Best model for total score and its application: GLM family (gaussian) link(power -0.3) (cubic splines, 3 knots). Supplementary Table 4. Best model with domains and its application: Fractional logistic (cubic splines, 3 knots). Supplementary Fig. 1. Distribution of: (a) EQ-5D-5L index and (b) CLDQ-NASH total scores in patients considered obese and not obese. CLDQ-NASH Chronic Liver Disease Questionnaire – Nonalcoholic Steatohepatitis. Supplementary Fig. 2. Distribution of: (a) EQ-5D-5L index and (b) CLDQ-NASH total scores in patients with and without T2D. CLDQ-NASH Chronic Liver Disease Questionnaire – Nonalcoholic Steatohepatitis; T2D type 2 diabetes. Supplementary Fig. 3. Predicting EQ-5D-5L from CLDQ-NASH domains: fractional logistic model (with cubic splines, 3 knots). Patient-recorded or actual values are shown in pink, the black dashed line represents the CLDQ-NASH derived values, and the blue dashed lines represent 95% confidence intervals of the predicted values. CLDQ-NASH Chronic Liver Disease Questionnaire – Nonalcoholic Steatohepatitis. [file 12955_2023_2195_MOESM1_ESM.docx]
